# Supplementary material for: A Global Screen for Assembly State Changes of the Mitotic Proteome by SEC-SWATH-MS
Source: Cell Syst. 2020 Feb 26;10(2):133–155.e6. doi: 10.1016/j.cels.2020.01.001 (PMC7042714; doi:10.1016/j.cels.2020.01.001)

O60231 | DHX16_HUMAN | DHX16 DBP2 DDX16 KIAA0577
Monomer MW [kDa]: 119.264 Monomer expected elution fraction: 39

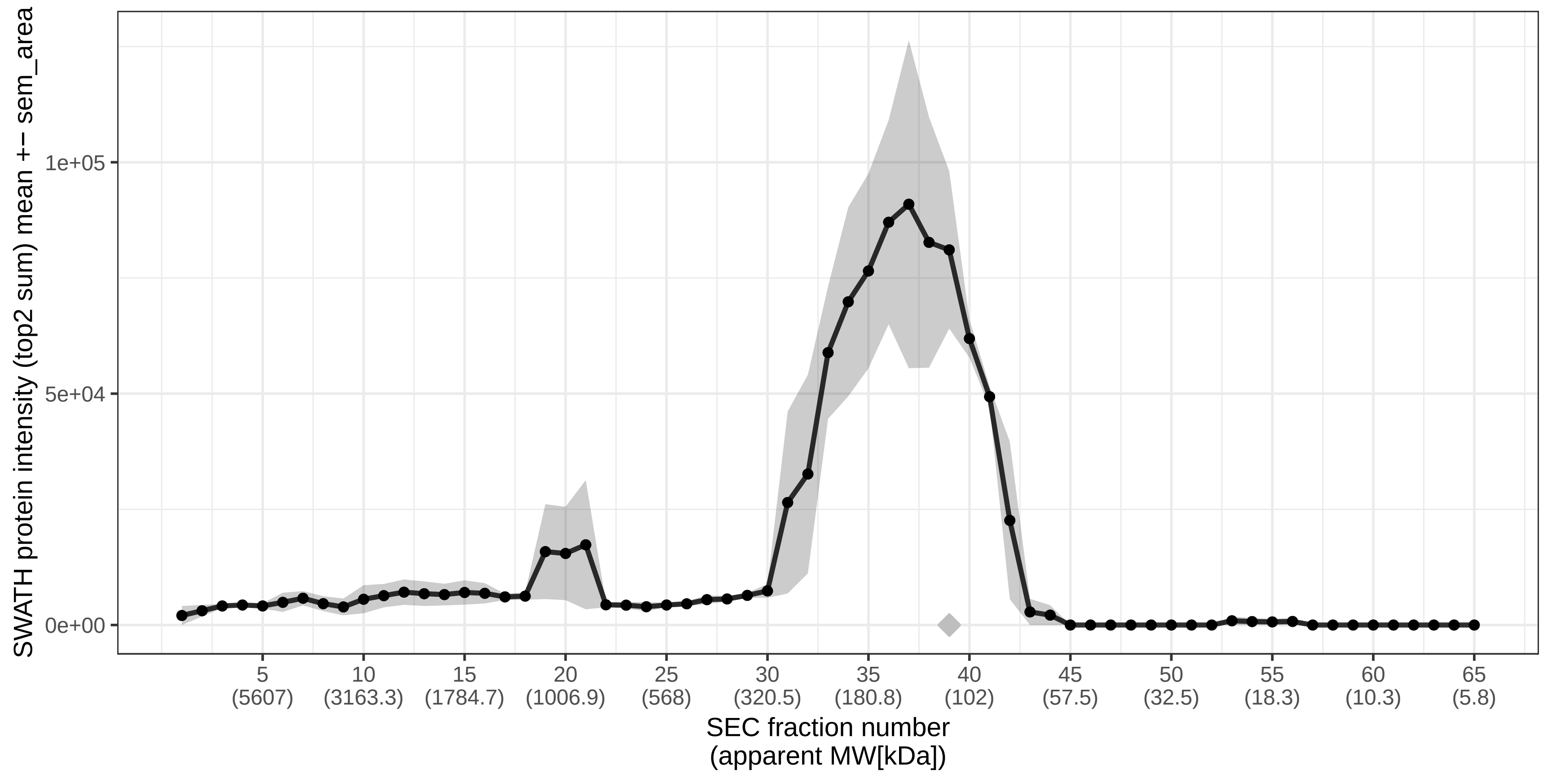

Supplement: Data S1. SEC-SWATH-MS Protein Chromatograms, Related to Figure 1 [file mmc6.zip › SECchrom_O60231_DHX16_HUMAN_DHX16_DBP2_DDX16_KIAA0577.pdf]
